# iEnhancer-DCSV: Predicting enhancers and their strength based on DenseNet and improved convolutional block attention module

**DOI:** 10.3389/fgene.2023.1132018

**Published:** 2023-03-01

**Authors:** Jianhua Jia, Rufeng Lei, Lulu Qin, Genqiang Wu, Xin Wei

**Affiliations:** ^1^ School of Information Engineering, Jingdezhen Ceramic University, Jingdezhen, China; ^2^ Business School, Jiangxi Institute of Fashion Technology, Nanchang, China

**Keywords:** enhancer, DenseNet, channel attention, spatial attention, ensemble learning

## Abstract

Enhancers play a crucial role in controlling gene transcription and expression. Therefore, bioinformatics puts many emphases on predicting enhancers and their strength. It is vital to create quick and accurate calculating techniques because conventional biomedical tests take too long time and are too expensive. This paper proposed a new predictor called iEnhancer-DCSV built on a modified densely connected convolutional network (DenseNet) and an improved convolutional block attention module (CBAM). Coding was performed using one-hot and nucleotide chemical property (NCP). DenseNet was used to extract advanced features from raw coding. The channel attention and spatial attention modules were used to evaluate the significance of the advanced features and then input into a fully connected neural network to yield the prediction probabilities. Finally, ensemble learning was employed on the final categorization findings *via* voting. According to the experimental results on the test set, the first layer of enhancer recognition achieved an accuracy of 78.95%, and the Matthews correlation coefficient value was 0.5809. The second layer of enhancer strength prediction achieved an accuracy of 80.70%, and the Matthews correlation coefficient value was 0.6609. The iEnhancer-DCSV method can be found at https://github.com/leirufeng/iEnhancer-DCSV. It is easy to obtain the desired results without using the complex mathematical formulas involved.

## 1 Introduction

Genes are functional areas of an organism’s DNA (Dai et al., 2018; Kong et al., 2020) that hold genetic information. The gene is transferred to the protein through a sequence of transcription (Maston et al., 2006) and translation (Xiao et al., 2016), and proteins control the organism’s exterior phenotypic shape (Buccitelli and Selbach, 2020). Transcription is one of the most crucial aspects of gene expression. The enhancer and promoter (Cvetesic and Lenhard, 2017) are the most significant sequence regions for transcriptional activity. An enhancer is a brief non-coding DNA fragment on DNA (Kim et al., 2010) and controls rapid and slow gene expression (Shrinivas et al., 2019). According to previous studies, several illnesses (Yang et al., 2022) are produced as a result of enhancer mutations and deletions (Emison et al., 2005; Liu G. et al., 2018; Boyd et al., 2018; Wu et al., 2019). In terms of the activities they express, the enhancers may be categorized into groups, such as strong and weak enhancers, closed (balanced) enhancers, and latent enhancers (Shlyueva et al., 2014). Therefore, understanding and recognizing these specific gene sequence segments is an urgent problem (Pennacchio et al., 2013).

Traditional medical experimental methods (Yang et al., 2020) in bioinformatics are costly and time-consuming. Therefore, it is crucial to develop computational techniques and derive some excellent predictors (Firpi et al., 2010; Fernández and Miranda-Saavedra, 2012; Erwin et al., 2014; Ghandi et al., 2014; Kleftogiannis et al., 2015; Lu et al., 2015; Bu et al., 2017; Yang et al., 2017). However, these techniques have limitations in the prediction of strong and weak enhancers. Liu et al. (2015) developed a predictor called iEnhancer-2L based on the support vector machine (SVM) algorithm and used the sequence pseudo-K-tuple nucleotide composition (PseKNC) approach to encode features. Afterward, machine learning-based methods were applied to the prediction of enhancers, such as SVM (Jia and He, 2016; He and Jia, 2017), RF (Singh et al., 2013; Wang et al., 2021), and XGBoost (Cai et al., 2021), and many excellent predictors have been created. However, a single machine learning classifier has obvious performance drawbacks. A predictor based on an ensemble learning model (Liu B. et al., 2018) was developed to address this problem, which generally has a significantly better performance. The ensemble learning model has diversity and complexity in feature processing. For instance, Wang C. et al. (2022) developed a predictor called Enhancer-FRL, which used 10 feature methods for feature coding. The manual creation of feature coding is a relatively difficult problem, and the presence of many complex feature coding types can lead to dimensional disasters. Furthermore, the effectiveness of conventional machine learning models depends on the extracted complex features. Consequently, the development of a predictor that requires only simple features is crucial.

Nowadays, deep learning is becoming increasingly popular. Nguyen et al. (2019) proposed the iEnhancer-ECNN model based on convolutional neural networks (CNNs). Niu et al. (2021) proposed a model called the iEnhancer-EBLSTM based on bi-directional long short-term memory (Bi-LSTM). They used one-hot and K-mers coding techniques to encode the enhancer sequences and then fed these features into the deep learning network to get relatively good prediction results. For example, in the iEnhancer-ECNN model, the ACC and MCC of enhancer recognition results were 0.769 and 0.537, and the ACC and MCC of enhancer strength prediction results were 0.678 and 0.368, respectively. However, there is a wide gap in prediction precision using a better deep learning model.

In deep learning networks, CNNs with more convolutional layers extract more advanced local features but lead to the problem of gradient disappearance and network degradation. To solve this problem, the residual neural network (ResNet) (Li et al., 2022) uses a short-circuit connection structure, which allows the convolutional layers to be connected several layers apart and can solve the problem of network degradation to some extent. However, the densely connected convolutional network (DenseNet) (Huang et al., 2010) has been enhanced based on ResNet. DenseNet extracts richer feature information by reusing the features of each previous layer, and it is more effective than ResNet. The attention model is also increasingly used, and the essence of the attention model is to focus on more useful feature information and suppress useless feature information. Convolutional block attention module (CBAM) (Zhang et al., 2022) can focus on more useful feature information from channel and spatial dimensions. The current computational method has the disadvantages of poor performance and complex features. For this purpose, we developed a new predictor called iEnhancer-DCSV. The predictor is conducted using a modified DenseNet and an improved CBAM attention module. The DenseNet framework makes it easier to extract more advanced features. Experimental results show that our model outperforms the existing models. The iEnhancer-DCSV model is currently the optimal choice for predicting enhancers and their strengths.

## 2 Materials and methods

### 2.1 Benchmark dataset

The benchmark dataset was created by Liu et al. (2015). They took the enhancer fragments from nine cell lines, removed 80% of the redundant sequences with the CD-HIT (Huang et al., 2010) and then calculated the ideal fragment length of 200 bp for each enhancer sequence to create the final dataset. The dataset is split into two sections: a training dataset for the model’s training and an independent test dataset for model testing. The independent test dataset is made up of 200 enhancer samples (with 100 strongly and 100 weakly enhancer samples) and 200 non-enhancer samples, whereas the training dataset is made up of 1,484 enhancer samples (with 742 strongly and 742 weakly enhancer samples) and 1,484 non-enhancer samples. All enhancer samples in the independent test dataset were different from the training dataset to guarantee that the samples are independent. The benchmark dataset is described in Table 1 and may be downloaded conveniently from the website: https://github.com/leirufeng/iEnhancer-DCSV.

**TABLE 1:** Specifics of the benchmark dataset.

Layer	Original dataset	Enhancer	Non-enhancer
First layer	Training dataset	1,484	1,484
	Testing dataset	200	200

	Original dataset	Strong enhancers	Weak enhancers
Second layer	Training dataset	742	742
	Testing dataset	100	100

### 2.2 Feature coding schemes

Two simple and effective coding techniques are used in this study: one-hot and NCP. Notably, these two coding techniques produce columns with a dimension of 200, so they can be feature-combined. For instance, an enhancer sequence with a length of 200 bp can obtain a 4 × 200 feature matrix and a 3 × 200 feature matrix after one-hot and NCP coding, respectively. Finally, combining these two matrices through feature fusion can yield a 7 × 200 feature matrix. In this study, the enhancer sequence is considered a gray image by the feature coding matrix. The 7 × 200 matrix is directly used as the original feature input.

#### 2.2.1 One-hot coding

**FIGURE 1:**
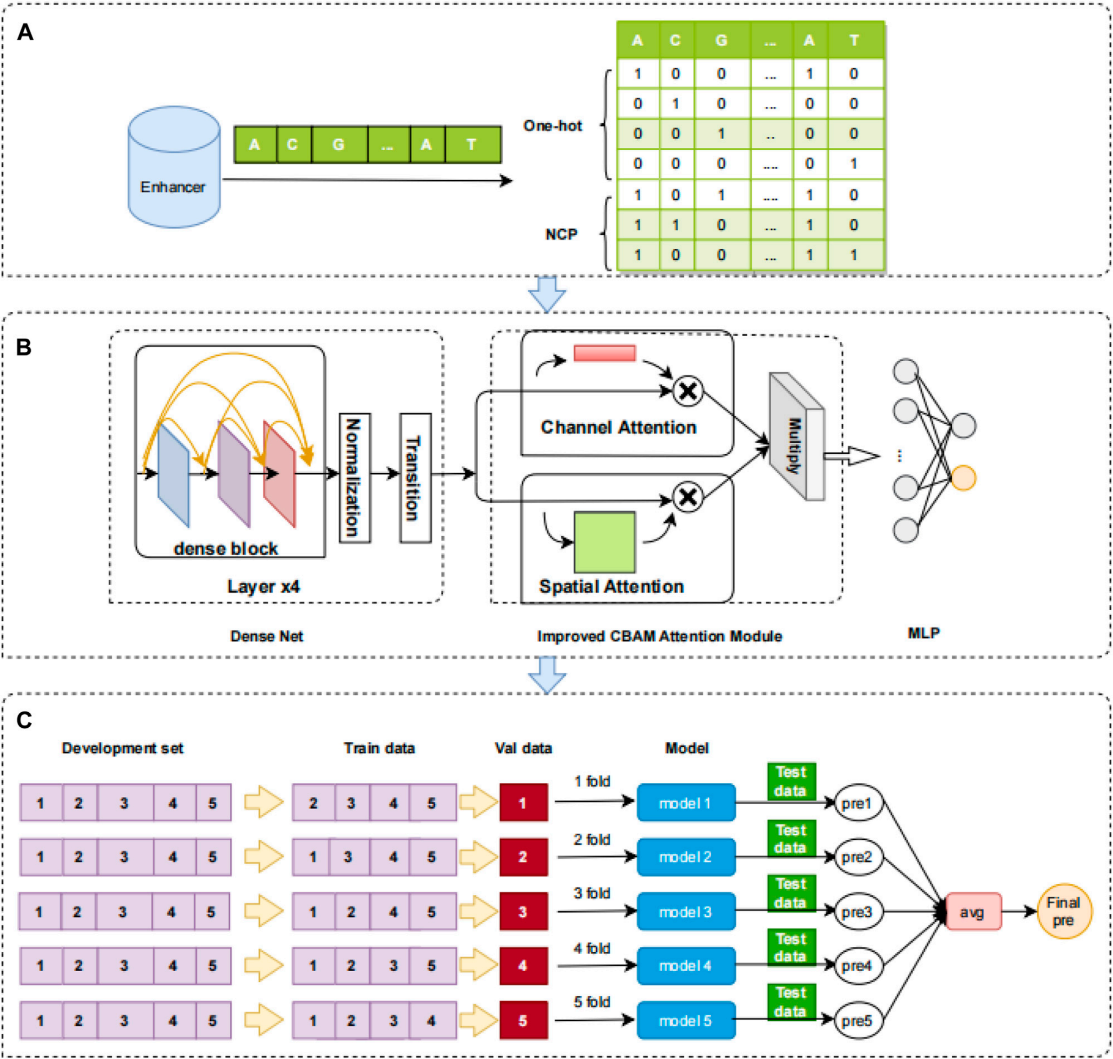
Overview of the iEnhancer-DCSV model. **(A)** Feature coding. One-hot and NCP are used to encode the enhancer sequence, and a 7 × 200 matrix is produced. **(B)** Framework of the iEnhancer-DCSV model. The original features are input directly to the modified DenseNet structure (which includes four dense blocks, normalized layers, and transition layers), and the improved structure is used to extract advanced features. Modules for spatial and channel attention are introduced to assess the extracted advanced features’ importance. The two evaluated advanced feature maps are multiplied together at the corresponding positions. The fully connected neural network is used to output the prediction probabilities. **(C)** Ensemble model. The model uses fivefold cross-validation, where each fold is tested using an independent test set, each test enhancer sequence generates five prediction probabilities, and the final classification is voted using ensemble learning.

In the field of bioinformatics, one-hot coding is one of the most used coding techniques. The advantages of this coding technique are its feasibility, efficiency, and ability to assure that each nucleotide letter is coded independently. The method is effective in avoiding the expression of interdependencies. This coding technique is particularly popular in bioinformatics. The double helix structure (Sinden et al., 1998) of DNA is widely known, and it is made up of four nucleotides: A (adenine deoxyribonucleotide), C (cytosine deoxyribonucleotide), G (guanine deoxyribonucleotide), and T (thymine deoxyribonucleotide) (Chou, 1984). The enhancer sequences are DNA sequences designated “0,1,2,3” in the order “ACGT.” The nucleotides in the sequences are then coded, and the coding length is four nucleotides. The coding elements are 0 and 1. The position corresponding to the nucleotide letter marker is coded as 1, and the other positions are coded as 0. For instance, “A” is coded as (1,0,0,0), “C” is coded as (0,1,0,0), “G” is coded as (0,0,1,0), and “T” is coded as (0,0,0,1) (Zhang et al., 2022). The one-hot coding is shown in Figure 1A.

#### 2.2.2 NCP coding

The four DNA nucleotides are structurally different from each other and have different chemical molecular structures (Zhang et al., 2022). For instance, C and T contain one loop each, whereas A and G have two loops between the four nucleotides. G and T may be classified as ketone groups from the standpoint of chemical composition, whereas A and C can be classified as amino groups. A and T have two hydrogen bonds, but C and G have three hydrogen bonds. The strength between C and G is more powerful than that between A and T. The specific chemical properties between nucleotides are shown in Table 2.

**TABLE 2:** Nucleotide chemical property.

Chemical property	Category	Nucleotide
Ring structure	Purine	A, G
	Pyrimidine	C, T
Functional group	Amino	A, C
	Keto	G, T
Hydrogen bonding	Strong	C, G
	Weak	A, T

Then, coding is performed based on the chemical characteristics. The nucleotide $N_{i}$ is located in position i in the sequence. Three chemical characteristics of nucleotide $N_{i}$ are “ring structure,” “functional group,” and “hydrogen bond strength” (Xiao et al., 2019). The vector representation of $N_{i}=\left(x_{i},y_{i},z_{i}\right)$ , $x_{i},y_{i},z_{i}$ is expressed as

$$\begin{array}{c} \{\begin{array}{c} \;x_{i}=\left\{\begin{array}{c} 1,if\;N_{i}\in \left\{A,G\right\},\\ 0,if\;N_{i}\in \left\{C,T\right\}, \end{array}\right.\\ y_{i}=\left\{\begin{array}{c} 1,if\;N_{i}\in \left\{A,C\right\},\\ 0,if\;N_{i}\in \left\{G,T\right\}, \end{array}\right.\\ \;z_{i}=\left\{\begin{array}{c} 1,if\;N_{i}\in \left\{A,T\right\},\\ 0,if\;N_{i}\in \left\{C,G\right\}. \end{array}\right. \end{array} \end{array}$$ (1)

A, C, G, and T may be encoded using this approach as (1,1,1), (0,1,0), (1,0,0), and (0,0,1). NCP coding is shown in Figure 1A.

### 2.3 Model construction

In this study, we constructed a network framework to automatically learn advanced features called iEnhancer-DCSV. The framework of iEnhancer-DCSV is divided into three parts: (A) feature coding, (B) framework of iEnhancer-DCSV model, and (C) ensemble model. The details are shown in Figure 1.

#### 2.3.1 DenseNet

In this study, we modified the initial DenseNet structure. The original DenseNet consists of a convolutional layer, a dense block layer, and a transition layer. First, convolution is applied to the original features. Then, the convolution features are processed by the dense block and transition layers. The dense block layer is a dense connection of all the preceding layers to the following layers. In particular, each layer accepts all its preceding layers as its additional input, enabling feature reuse. The transition layer, which mainly connects two adjacent dense blocks, reduces the feature map size. Instead, we deleted the first convolutional layer and added a batch normalization layer between the dense block layer and the transition layer. This processing method can extract better-quality feature information and reduce the risk of overfitting.

##### 2.3.1.1 Dense block

**FIGURE 2:**
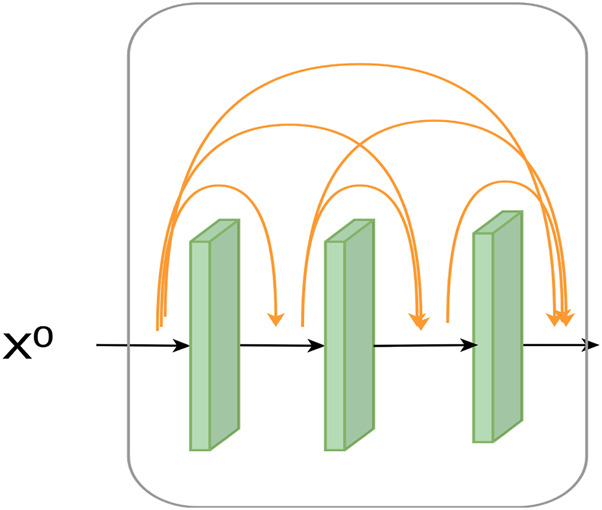
Structure of a dense block.

The traditional CNN network does not perform very well in extracting feature information. A convolutional structure called dense convolutional block extracts richer feature information by reusing previous features. Experimentally, the dense convolutional network feature extraction is proven better than traditional CNN. The structure diagram is shown in Figure 2.

In the dense block, the input of layer i is related to not only the output of layer i−1 , but the output of all the previous layers. The $X_{l}$ level is represented as follows:

$$\begin{array}{c} X_{l}=H_{l}\;\left(\left[X_{0}{,X}_{1}{,X}_{2},\ldots ,X_{l-1}\right]\right), \end{array}$$ (2)

where is denoted as layers $X_{0}$ to $X_{l-1}$ stitched together by the dimension of the channel. *H* is a non-linear combinatorial function. It is a combination of batch normalization, ReLU activation function, and convolution (3 × 3).

In this study, we used four dense blocks, each containing three layers of convolution. The final extraction of features was $X^{\left(seq\right)}$ .

##### 2.3.1.2 Transition layer

The l -1 layers in front of the dense block are combined by channel dimension. As the number of channels in the l -layer becomes larger, it leads to an explosion of parameters, along with a slow training speed. We can improve the efficiency by connecting a transition layer with the dense block layer. The transition layer consists of a 1 × 1 convolution and a 2 × 2 average pooling. It is a function of reducing the number of channels and parameters in the dense block layer by downsampling to compress the model.

#### 2.3.2 Batch normalization

Gradient explosion and gradient disappearance are serious problems in deep learning training, and this phenomenon tends to occur more likely in the deeper network structure. If the shallow parameters are changed, their fluctuations during backpropagation may be significant, resulting in significant variable shifts in the deeper network. Batch normalization (Min et al., 2016) has been shown to improve the generalization ability of the model. The batch normalization is expressed as follows:

$$\begin{array}{c} \tilde{x_{i}}=\frac{x_{i}-\mu }{\sqrt{\sigma^{2}-\epsilon }} \end{array},$$ (3)

$$\begin{array}{c} y_{i}=\gamma \tilde{x_{i}}+\beta , \end{array}$$ (4)

where A is the set of the feature dataset $\left[x_{1},x_{2},\ldots ,x_{i}\right]$ , μ is the mean of dataset A , and $\sigma^{2}$ is the variance of dataset A . γ and β are trainable parameters.

#### 2.3.3 Improved CBAM attention module

The CBAM attention module comprises channel attention and spatial attention modules (Chen et al., 2017). First, we use the channel attention module to evaluate the original features. Second, we take the feature map output from the channel attention module and feed it back into the spatial attention module. Finally, we output the final feature maps from the spatial attention module. This serial connection of CBAM attention modules has the disadvantage that the attention modules are all computed in a specific way, and the computation of weights destroys the feature shape of the input. This leads to inaccurate weight calculation of the spatial attention modules and loss of channel weighting information in the final feature map. We change the original serial approach in the CBAM attention module to a parallel method. The principle is to input the original features into the channel attention module and the spatial attention module and let the output features be multiplied by their corresponding positions. By this method, the effect of each attention model after evaluation can be maximally preserved and the expressiveness of the features can be improved.

##### 2.3.3.1 Channel attention module

**FIGURE 3:**
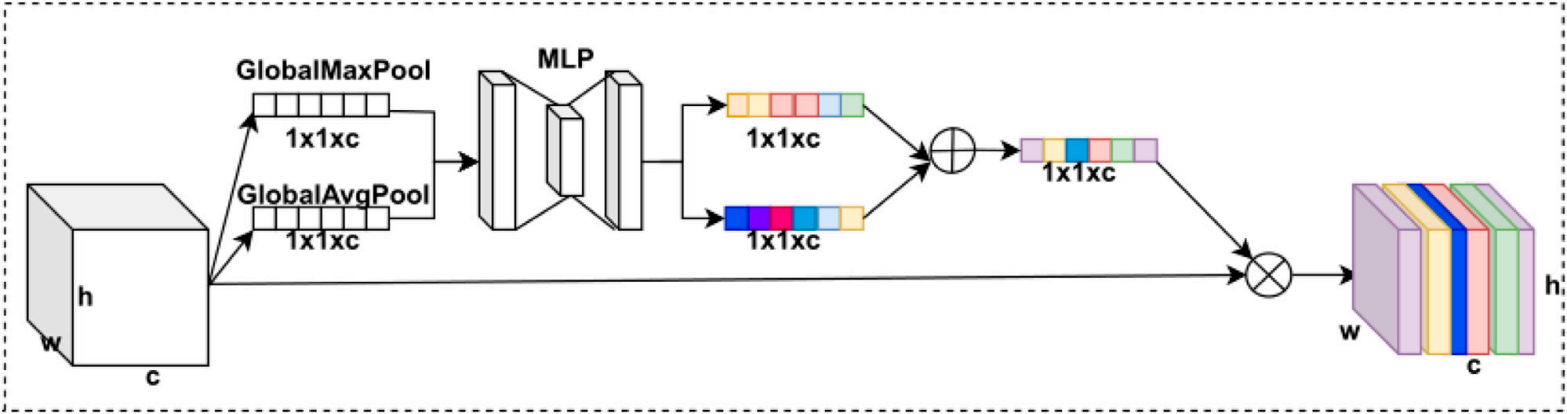
CBAM’s channel attention module structure.

In deep learning, the degree of importance varies between different feature map channels, so we use the channel attention module to calculate different weights for each channel. By weighting each channel of the feature map, the model automatically pays attention to the more useful channel information to achieve the fixation of channel dimension and compression of spatial dimension. The channel attention module comprises the max pooling layer, the average pooling layer, the MLP module, and the sigmoid activation function. The CBAM’s channel attention module structure is shown in Figure 3.

The channel attention module starts with the feature map passing through two parallel max pooling and average pooling layers, which are input into the fully connected neural network (MLP) module separately. Second, the two results of the MLP output are summed element by element, and the channel attention module weights are obtained using the sigmoid activation function. Finally, these weights are multiplied by the feature map to obtain the feature map of the channel attention model weighting. The CBAM’s channel attention model is expressed as follows:

$$\begin{array}{c} M_{c}\left(F\right)=\sigma \left(MLP\left(AvgPool\left(F\right)\right)+MLP\left(MaxPool\left(F\right)\right)\right), \end{array}$$ (5)

$$\begin{array}{c} F=F_{scale}\left(F,Mc\left(F\right)\right)=Mc\left(F\right)∙F, \end{array}$$ (6)

where pooling here is the global max pooling and the global average pooling. $F_{scale}\left(F,Mc\left(F\right)\right)$ denotes each channel-specific value of F multiplied by the weight $Mc\left(F\right)$ .

##### 2.3.3.2 Spatial attention module

**FIGURE 4:**
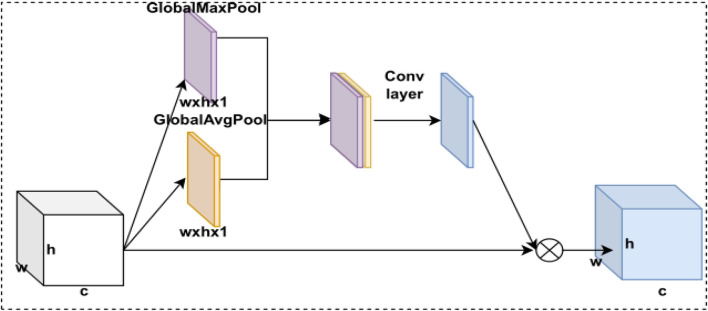
CBAM’s spatial attention module structure.

In deep learning, different receptive fields have different degrees of value to the feature map, so we use a spatial attention model to calculate the weights between receptive fields. By weighting the receptive fields, we allow the model to focus on the more useful target location information to achieve a constant spatial dimension and a compressed channel dimension. The spatial attention model is implemented through a max pooling layer, an average pooling layer, a CNN module, and a sigmoid activation function. The CBAM’s spatial attention module structure is shown in Figure 4.

The spatial attention model first passes the feature maps through two parallel max pooling and average pooling layers and performs a stitching operation on the two pooling feature maps. Then, the newly obtained features are input into the CNN module to be transformed into a feature map with channel number 1, and the spatial attention module weights are obtained by the sigmoid activation function. Finally, this weight is multiplied by the feature map to obtain the weighted feature map of the spatial attention model. The CBAM’s spatial attention model is expressed as follows:

$$\begin{array}{c} M_{s}\left(F\right)=\sigma \left(f^{7\times 7}\left(\left[AvgPool\left(F\right);MaxPool\left(F\right)\right]\right)\right), \end{array}$$ (7)

$$\begin{array}{c} F=F_{scale}\left(F,Ms\left(F\right)\right)=Ms\left(F\right)∙F. \end{array}$$ (8)

Pooling here is the global max pooling and global average pooling. The size of the convolutional kernel used in the CNN module is 7 × 7. Finally, $F_{scale}\left(F,Mc\left(F\right)\right)$ denotes each receptive field of F multiplied by the weight $Mc\left(F\right)$ .

#### 2.3.4 Fully connected neural network

We used a fully connected neural network (Wang. et al., 2022b) to predict the enhancers and their strength. After we extracted the advanced features, the size of the advanced features was reduced using a pooling layer. Then, these features are flattened into vectors, which are later input into the fully connected neural network. Finally, the softmax function is used to calculate the predicted probability of the enhancers. The softmax formula is expressed as

$$\begin{array}{c} P\left(y=i|x\right)=\frac{e^{W_{i}^{s}*X}}{\sum_{j=1}^{C}e^{W_{j}^{s}*X}} \end{array},$$ (9)

where $W_{i}^{s}$ and $W_{j}^{s}$ denote the weights in the fully connected neural network, X denotes the sample, and C is the number of categories. $P\left(y=i|x\right)$ denotes the probability that x is predicted to be i . This is a dichotomous problem, i = 0 or i = 1.

#### 2.3.5 Ensemble model

There is an ensemble method called bagging (Bauer and Kohavi, 1999). It is accomplished by training several different models, allowing independent test data to calculate the predicted results using different models and then averaging them. This ensemble learning approach is called model averaging. The advantage of model averaging is that different models do not usually produce the same error on the test data, and it is a very powerful method for reducing generalization errors.

In this study, we used a fivefold cross-validation method (Shang et al., 2022). The training dataset was divided into five parts: four for training and one for validation. We used an independent test set put into each fold in cross-validation, by which five predictions are obtained. Finally, the final prediction results are obtained by the voting method. The ensemble method is shown in Figure 1C.

### 2.4 Performance evaluation

Scientific evaluation metrics are a measure of model performance. In this study, the evaluation of model performance contains four metrics: sensitivity (Sn), specificity (Sp), accuracy (Acc), and Mathew’s correlation coefficient (MCC) (Sokolova and Lapalme, 2009). The specific calculation formula is shown as follows:

$$\left\{\begin{array}{l} \;Sp=\frac{TN}{TN+FP},\\ \;Sn=\frac{TP}{TP+FN},\\ \;Acc=\frac{TP+TN}{TP+TN+FP+FN},\\ \;MCC=\frac{TP\times TN-FP\times FN}{\sqrt{\left(TP+FP\right)\times \left(TP+FN\right)\times \left(TN+FP\right)\times \left(TN+FN\right)}}, \end{array}\right.$$ (10)

where TP, TN, FP, and FN are the four metrics in the confusion matrix, representing true positive, true negative, false positive, and false negative, respectively (Niu et al., 2021). In addition, we added the ROC curve area AUC metric (Vacic et al., 2006) to evaluate the model, and higher values of these metrics indicate better model performance.

## 3 Results and discussion

### 3.1 Construction of the first layer (enhancer recognition) model

The recognition of enhancers in the first layer is very important to complete the prediction mission. For the first layer of enhancer recognition, we used the iEnhancer-DCSV network framework. The advanced feature extraction and weight assignment are performed automatically by the model’s iEnhancer-DCSV network framework. First, the enhancer sequences are encoded using the one-hot and NCP methods, and then feature coding is fed into the DenseNet to extract advanced features. These advanced features are input into the channel attention module and the spatial attention module, respectively. The two evaluated advanced feature maps are multiplied at the corresponding positions, and then the pooling layer is used to compress the feature size. Finally, a fully connected neural network is used to derive the predicted probabilities. We validate the model by putting independent test sets into each fold of the fivefold cross-validation. The aforementioned five-time results are passed through a soft voting mechanism to arrive at the final prediction. The whole process was cycled 10 times to verify the stability of the model, and the obtained individual performance metrics were averaged. The experimental results for SN, SP, Acc, and MCC were 80.25%, 77.65%, 78.95%, and 0.5809, respectively.

### 3.2 Construction of the second layer (strong and weak enhancer prediction) model

On the basis of the correct identification of enhancers in the first layer, the second layer predicts the strengths and weaknesses of enhancers. As the second layer has less training data and the complex network structure can lead to overfitting, we removed the attention module from the iEnhancer-DCSV network framework and used the same training as the first layer, with experimental results of 99.10%, 62.30%, 80.70%, and 0.6609 for SN, SP, Acc, and MCC, respectively.

### 3.3 Comparison of different coding methods

Currently, feature engineering has been a very important part of the process because building a model and using a simple and efficient coding method is crucial. In this study, we compared the one-hot + NCP coding, one-hot coding, and NCP coding to determine the final coding method. We input the three encoding methods into the two network frameworks, layer 1 and layer 2, respectively, and the results of the experiment are shown in Table 3. In the first layer (enhancer recognition), the one-hot + NCP coding was slightly better than the one-hot coding and better than the NCP coding. In the second layer (strong and weak enhancer prediction), the one-hot + NCP coding was much better than these two coding types. Therefore, we adopted one-hot + NCP coding as the final coding method in this study.

**TABLE 3:** Comparison results of different coding schemes.

Layer	Coding	SN (%)	SP (%)	Acc (%)	MCC	AUC
First layer	One-hot	81.70	75.50	78.60	0.5737	0.8275
	NCP	83.25	70.50	76.88	0.5428	0.8168
	One-hot + NCP	80.25	**77.65**	**78.95**	**0.5809**	**0.8527**
Second layer	One-hot	60.30	72.80	66.55	0.3418	0.7491
	NCP	90.50	53.40	71.95	0.4780	0.7666
	One-hot + NCP	**99.10**	62.30	**80.70**	**0.6609**	**0.8686**

### 3.4 Comparison of different model frameworks

In this study, we used six network frameworks: ResNet, DenseNet, DenseNet + channel attention model, DenseNet + spatial attention model, DenseNet + CBAM attention model, and DenseNet + improved CBAM attention model. We tested these five network frameworks in the first layer (enhancer recognition) task because the amount of data for the second layer (enhancer strength prediction) task was too small. The original features were extracted using each of these five network frameworks for the high-level features, and the best-performing network framework was selected based on the experimental results. The experimental comparison results are shown in Table 4. Adding an attention model behind the DenseNet is already very effective, and the improved CBAM attention model integrates the advantages of both attention models. However, the improved effect is limited because the shape of the feature map is too small. The results show that the DenseNet + improved CBAM attention network framework works better. Therefore, we finally chose the DenseNet + improved CBAM attentional network framework model.

**TABLE 4:** Comparison with different architecture methods at layer 1 (enhancer recognition).

Model framework	SN (%)	SP (%)	Acc (%)	MCC	AUC
ResNet	69.80	77.90	73.85	0.4927	0.8211
DenseNet	83.10	68.50	75.80	0.5219	0.8108
DenseNet + channel attention	78.20	78.35	78.27	0.5686	0.8316
DenseNet + spatial attention	78.75	78.20	78.48	0.5717	0.8304
DenseNet + CBAM attention	83.70	67.25	75.48	0.5183	0.8046
DenseNet + improved CBAM attention	80.25	77.65	**78.95**	**0.5809**	**0.8527**

### 3.5 Performance of iEnhancer-DCSV on the training dataset

**FIGURE 5:**
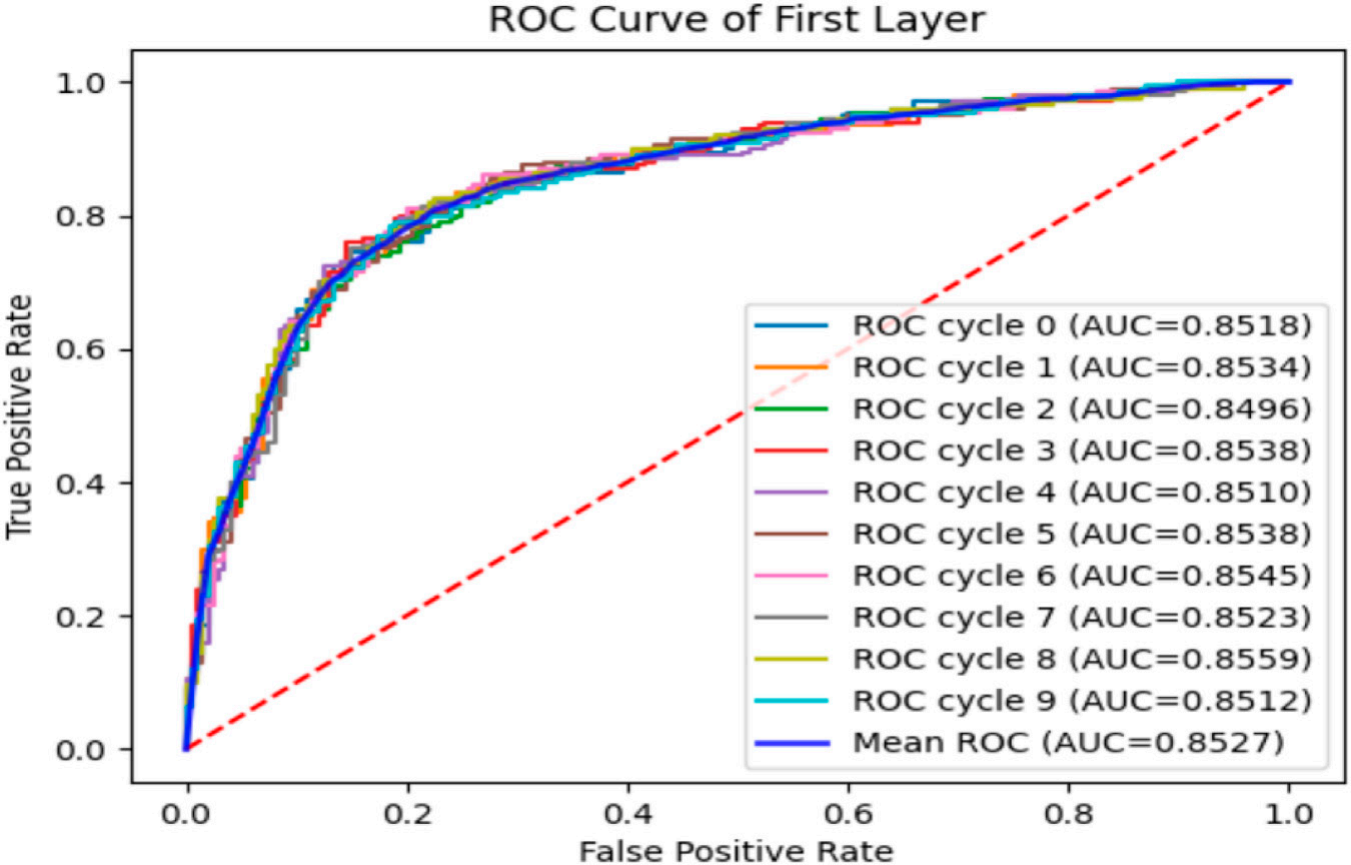
ROC curves for layer 1 (enhancer recognition).

**FIGURE 6:**
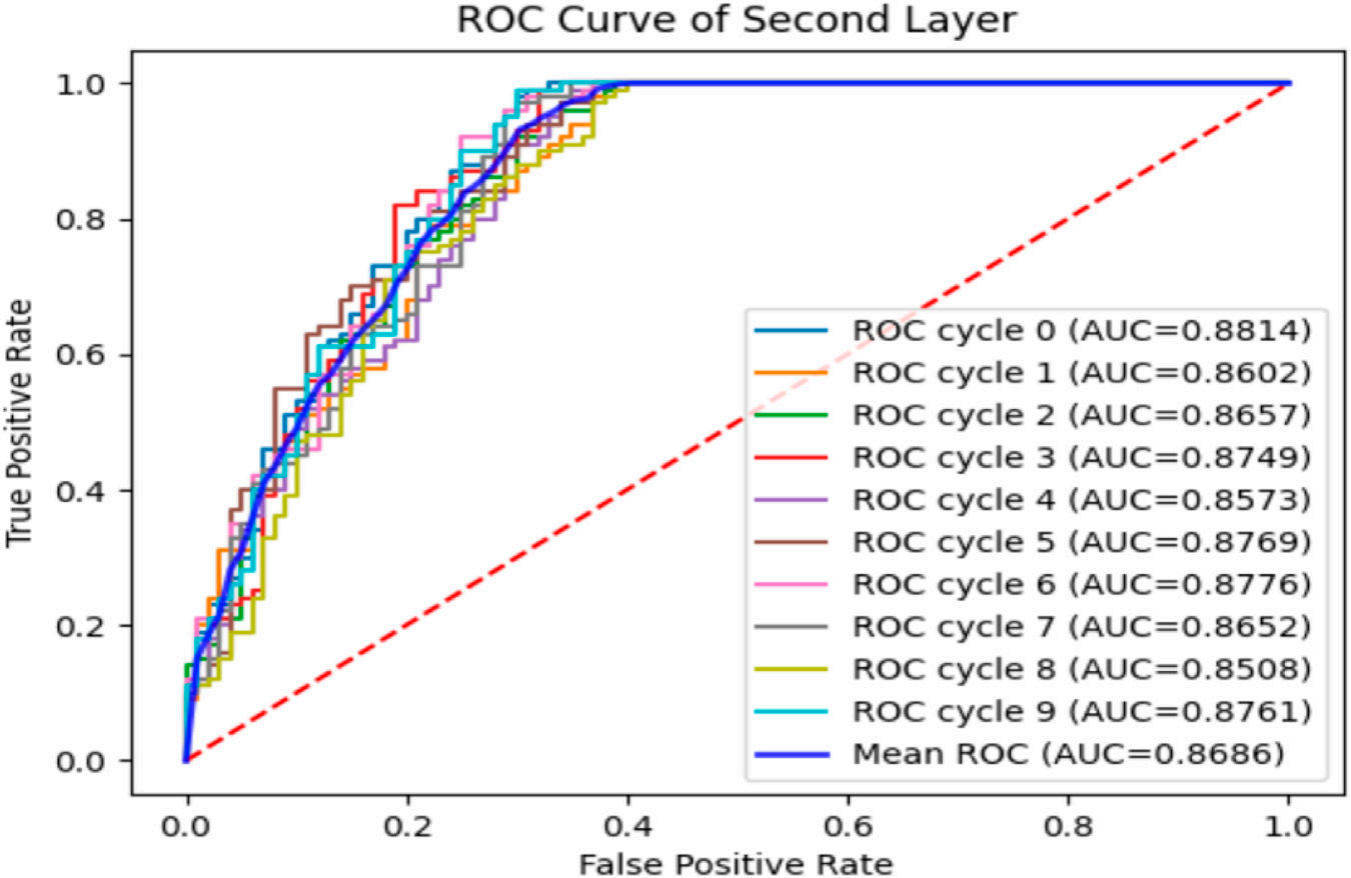
ROC curves for layer 2 (enhancer strength prediction).

To verify the performance of the iEnhancer-DCSV classifier, we cycled through 10 times of fivefold cross-validation, and the experimental results are shown in Table 5. We found that the values of the evaluation metrics fluctuated relatively steadily on the first (enhancer recognition) and second (enhancer strength prediction) layer tasks, indicating that the iEnhancer-DCSV model has good generalization capability. Figure 5 shows the ROC curves of the first layer (enhancer recognition) with a mean AUC value of 0.8527 in 10 experiments, and Figure 6 shows the ROC curves of the second layer (enhancer strength prediction) with a mean AUC value of 0.8686 in 10 experiments. The results show that our proposed iEnhancer-DCSV has good performance.

**TABLE 5:** Performance of iEnhancer-DCSV in 10 trials.

Layer	Cycle index	Sn (%)	Sp (%)	Acc (%)	MCC
First layer	0	78.50	78.00	78.25	0.5650
	1	83.00	75.50	79.25	0.5866
	2	80.50	75.00	77.75	0.5558
	3	74.00	85.50	79.75	0.5989
	4	77.00	81.50	79.25	0.5855
	5	87.50	68.00	77.75	0.5658
	6	80.50	80.00	80.25	0.6050
	7	79.50	80.00	79.75	0.5950
	8	80.50	78.00	79.25	0.5851
	9	81.50	75.00	78.25	0.5661
	Mean ± STD	**80.25 ± 3.39**	**77.65 ± 4.47**	**78.95 ± 0.84**	**0.5809 ± 0.0158**
Second layer	0	99.99	61.99	81.00	0.6702
	1	95.99	62.99	79.50	0.6250
	2	95.99	64.99	80.50	0.6416
	3	99.99	60.99	80.50	0.6624
	4	99.99	56.99	78.50	0.6313
	5	99.99	61.99	81.00	0.6702
	6	99.99	60.99	80.50	0.6624
	7	99.99	64.99	82.50	0.6938
	8	99.99	58.99	79.50	0.6468
	9	98.99	67.99	83.50	0.7047
	Mean ± STD	**99.10 ± 1.58**	**62.30 ± 3.00**	**80.70 ± 1.38**	**0.6609 ± 0.0243**

### 3.6 Comparison of iEnhancer-DCSV with existing predictors

The iEnhancer-DCSV predictor proposed in this study is compared with seven existing predictors. The performance of independent datasets under different methods is shown in Table 6. The iEnhancer-DCSV predictor has better Acc and MCC metrics compared with others. The improvement ranges for ACC and MCC in the first layer (enhancer recognition) were 1.95%–5.95% and 0.0202–0.1205, respectively, and the improvement ranges for ACC and MCC in the second layer (enhancer strength prediction) were 7.2%–25.7% and 0.1218–0.5588, respectively. Meanwhile, in the first and second layers, the SN and SP metrics also have some advantages, indicating that iEnhancer-DCSV is more balanced and has more stable and superior performance in identifying positive and negative samples. The iEnhancer-DCSV predictor is expected to be the most advanced and representative tool for predicting enhancement and its strengths and weaknesses.

**TABLE 6:** Comparison with other methods on the same independent datasets.

Layer	Predictor	SN	SP	Acc	MCC	AUC	Source
First layer	iEnhancer-2L	71.00	75.00	73.00	0.4604	0.8062	Liu et al. (2015)
	EnhancerPred	73.50	74.50	74.00	0.4800	0.8013	Jia and He (2016)
	iEnhancer-EL	71.00	78.50	74.75	0.4964	0.8173	Liu et al. (2018a)
	iEnhancer-ECNN	78.50	75.20	76.90	0.5370	0.8320	Nguyen et (2019)
	iEnhancer-XG	75.75	74.00	77.50	0.5150	—	Cai et al. (2021)
	iEnhancer-EBLSTM	75.50	79.50	77.20	0.5340	0.7720	Niu et al. (2021)
	Enhancer-FRL	80.50	75.50	78.00	0.5607	0.8573	Wang et al. (2022a)
	iEnhancer-DCSV	80.25	77.65	**78.95**	**0.5809**	0.8527	This study
Second layer	iEnhancer-2L	47.00	74.00	60.50	0.2181	0.6678	Liu et al. (2015)
	EnhancerPred	45.00	65.00	55.00	0.1021	0.5790	Jia and He (2016)
	iEnhancer-EL	54.00	68.00	61.00	0.2222	0.6801	Liu et al. (2018b)
	iEnhancer-ECNN	79.10	56.40	67.80	0.3680	0.7480	Nguyen et (2019)
	iEnhancer-XG	70.00	57.00	63.50	0.2720	—	Cai et al. (2021)
	iEnhancer-EBLSTM	81.20	53.60	65.80	0.3240	0.6580	Niu et al. (2021)
	Enhancer-FRL	98.00	49.00	73.50	0.5391	0.8723	Wang et al. (2022b)
	iEnhancer-DCSV	**99.10**	62.30	**80.70**	**0.6609**	0.8686	This study

## 4 Conclusion

In this study, we propose a new predictor of enhancer recognition and its strength called iEnhancer-DCSV. It is based on DenseNet and an improved CBAM attention module approach. The experimental results demonstrate that the MCC value for enhancer identification on the independent test set is 0.5809, and the MCC value for enhancer strength prediction is 0.6609. This indicates that the iEnhancer-DCSV predictor has good performance and generalization ability, which is better than the existing prediction tools. We combine deep learning methods with enhancer research to innovate computational methods in the field of bioinformatics and enrich enhancer research. In the future, the iEnhancer-DCSV predictor not only is applicable to enhancer classification tasks but can also be used in different prediction tasks, making its use convenient for researchers.

Of course, some deficiencies must be overcome in our proposed model. The current enhancer sample of data is small and fails to sufficiently promote the performance of the iEnhancer-DCSV model using a big data-driven approach. In addition, data enhancement strategies were not employed to augment our data samples, such as generative adversarial networks (GANs) (Li and Zhang, 2021). This will be our future work issue to address. However, as the research on enhancers progresses, the disadvantage of a small amount of data will gradually disappear, and better deep learning methods will be used in the research, creating more possibilities for future enhancer recognition and strength prediction.

## Data Availability

The original contributions presented in the study are included in the article/Supplementary Material, further inquiries can be directed to the corresponding authors.
